# Bodily Experience in Schizophrenia: Factors Underlying a Disturbed Sense of Body Ownership

**DOI:** 10.3389/fnhum.2016.00305

**Published:** 2016-06-17

**Authors:** Maayke Klaver, H. Chris Dijkerman

**Affiliations:** Department of Experimental Psychology, Helmholtz Institute, Utrecht UniversityCS Utrecht, Netherlands

**Keywords:** body ownership, self-agency, schizophrenia, multisensory integration, body representations, internal predictions, temporal binding

## Abstract

Emerging evidence is now challenging the view that patients diagnosed with schizophrenia experience a selective deficit in their sense of agency. Additional disturbances seem to exist in their sense of body ownership. However, the factors underlying this disturbance in body ownership remain elusive. Knowledge of these factors, and increased understanding of how body ownership is related to other abnormalities seen in schizophrenia, could ultimately advance development of new treatments. Research on body ownership in schizophrenia has mainly been investigated with the rubber hand illusion (RHI). Schizophrenia patients show higher susceptibility to the RHI, which may be explained by a stronger reliance on multisensory information over weaker stored body representations. This review shows that a coherent sense of body ownership arises from the integration of both bottom-up sensory processes and higher order, top-down bodily- and perceptual representations. Multisensory integration, temporal binding, anticipation, intention and efferent signals all partly modulate the complex experience of body ownership. Specifically, we propose that patients with schizophrenia have weaker stored body representations, and rely to a greater extent on external stimuli, such as visual information, due to imprecise or highly variable internal predictions. Moreover, the reduced sense of agency in schizophrenia may additionally contribute to the disturbed sense of body ownership, as evidence from healthy participants suggests that agency and body ownership are interrelated. *Vice versa*, a reduced sense of body ownership may also contribute to a reduced sense of agency. Future studies should explicitly target the precise relationship between the two in schizophrenia.

## Introduction

Schizophrenia is described as a psychiatric disorder in which there is loss of coherence in the minimal sense of self (Nelson et al., 2014). From a phenomenological perspective, three layers in the sense of self are distinguished (Sass and Parnas, 2003). At the first and most basic level, there is an implicit and unconscious awareness of oneself as an origin of experience, as a medium for relating to the world, referred to the “minimal self” or *ipseity*. Secondly, a more explicit or reflective awareness of the self exists as a constant subject of experience and action. Finally, the “social” or “narrative self” refers to personal characteristics such as habits, social identity and history, and often involves reflective, metacognitive processes (Nelson et al., 2014). These different levels of self-experience are interconnected and cannot be observed in isolation from each other.

Different authors have stated that the most basic level of self-experience, *ipseity*, is rooted in the bodily experience (e.g., Merleau-Ponty, 1945; Piaget, 1954). The sense of one’s own body is variously termed “corporeal awareness”, “bodily self-consciousness”, or “embodiment”, and is described as an implicit form of knowledge that is both non-conceptual and somatic (Kant and Smith, 2003). Embodiment specifically is defined as* “the perception that one’s sense of self is localized within one’s bodily borders”* (Arzy et al., 2006). Some authors have proposed that this embodiment is a necessary prerequisite for other kinds of self-experience (e.g., Kant and Smith, 2003). According to the phenomenological approach, the coherence between the diverse symptoms in schizophrenia can only be understood if their shared bodily roots are taken into account (de Haan and Fuchs, 2010).

The embodied sense of self is a complex phenomenon that consists of different components, including body ownership and the sense of agency over actions. In this review, we adopt a broad definition of body ownership conceived by Tsakiris et al. (2007) as *“the special perceptual status of one’s own body, which makes bodily sensations seem unique to oneself”.* In healthy people, a sense of body ownership is continuously present and they therefore experience body ownership not only when performing intended actions, but also when resting and during passive movement. The sense of agency, on the other hand, is the perception that one is the initiator of one’s actions. The observation that people with schizophrenia often misattribute their own actions to someone or something else, or deny intending their actions, has led to the prevailing idea that there is a selective impairment in their sense of agency, while body ownership is thought to be intact.

This prevalent view, in which patients with schizophrenia have a disturbed sense of agency but a normal sense of body ownership, has caused research to focus on motor control processes. To date, only few studies have specifically examined body ownership in schizophrenia, mainly with the use of the rubber hand illusion (RHI) paradigm, first described by Botvinick and Cohen (1998). During this illusion, the participant’s hand is hidden from view whilst a rubber model hand is placed in front of him. The experimenter simultaneously strokes the rubber hand and the participant’s hidden hand with two brushes. This produces an illusory sensation of ownership over the rubber hand and a shift in perceived hand location toward the fake hand (Botvinick and Cohen, 1998).

This RHI is generally experienced more intensely in people with schizophrenia (Peled et al., 2000, 2003; Thakkar et al., 2011; Germine et al., 2013). This finding challenges the idea that people with schizophrenia have a selective deficit in sense of agency. Additional disturbances in the sense of body ownership seem to exist. However, the underlying factors contributing to a disturbed sense of body ownership in schizophrenia are not clear. We will therefore review current evidence, and examine which factors contribute to a disturbed sense of body ownership in people diagnosed with schizophrenia. Specific studies and experiments that have contributed to an increased understanding of bodily experience will be highlighted. Finally, the connection between agency and ownership will be investigated. A summary of the possible factors underlying a disturbed sense of body ownership in schizophrenia is included in Table 1. Knowledge on how body ownership is related to other types of self-experience and psychosis-proneness can help increase our understanding of factors that lead to psychosis development and could ultimately advance development of new treatments.

**Table 1:** **The different factors possibly underlying a disturbed sense of body ownership in schizophrenia**.

Factor		Literature
Multisensory integration	A stronger effect of visual information overriding weaker multisensory integration.	Peled et al. (2000, 2003), Asai et al. (2011), Thakkar et al. (2011) and Germine et al. (2013)
Temporal and intentional binding	A “longer” window of temporal binding, in which events that happen further away in time are experienced as co-occurring.	Elvevåg et al. (2003), Franck et al. (2005), Foucher et al. (2007) and Graham et al. (2014)
Predictive mechanisms and anticipation	More variable predictive mechanisms resulting in a higher reliance on external information such as vision.	Ross et al. (2000), Voss et al. (2010), Lalanne et al. (2012) and Ferri et al. (2014)
Efferent motor signals	Aberrant efferent signals possibly leading to a more flexible sense of body ownership and higher interference of external information on the self.	Malenka et al. (1982), Frith and Done (1989), Daprati et al. (1997), Blakemore et al. (2000, 2002); Shergill et al. (2005) and Synofzik et al. (2010)
Self-agency	Agency and ownership are dissociable components of self-experience, but they do seem to interact. The disturbed sense of agency may therefore contribute to a disturbed sense of ownership in schizophrenia.	Tsakiris et al. (2006, 2010a), Longo et al. (2008), Waters and Badcock (2010), Kalckert and Ehrsson (2012, 2014), Asai (2015), Louzolo et al. (2015), and Garbarini et al. (2016)

*Only research specifically targeted at schizophrenia patients is included except for self agency, in which also evidence from healthy individuals is taken into account*.

## Multisensory Integration

Multisensory integration involves the processing of sensory input from various modalities and the resolution of possible conflicts in order to represent the body and the world coherently. Body ownership specifically is thought to result from the integration of visual and proprioceptive signals (Giummarra et al., 2008). The RHI depends on an interaction between sensory inputs from three different modalities, namely vision, touch, and proprioception. In this paradigm, a distorted sense of body ownership can be elicited by inducing a sensory conflict. Seeing the tactile stimulation on the rubber hand, while simultaneously feeling this on one’s own hand, results in a visual capture of the tactile sensation which subsequently causes a feeling of relocalization of one’s own hand towards the location of the rubber hand. This is termed “proprioceptive drift” and is thought to result from the dominance of vision over proprioception (Botvinick and Cohen, 1998). Using a psychometric approach with principal component analysis, Longo et al. (2008) found four dissociable components of the RHI experience, namely embodiment of the rubber hand, loss of the own hand, movement, and affect. Embodiment could in turn be separated into the three tightly interacting components of agency, ownership and location. Ownership specifically correlated with proprioceptive drift (Longo et al., 2008). This is in line with results obtained by Holmes et al. (2006), who found that proprioceptive bias correlated to questionnaire items investigating specifically ownership, but not other types of questions (Holmes et al., 2006).

Strength of the RHI has traditionally been measured in three ways, namely with the use of introspective questionnaires, proprioceptive drift and skin conductance response elicited by threatening or injuring the rubber hand. Statements on a questionnaire cannot be used to quantify the illusion in absolute terms, but should rather be used to measure differences between conditions. Indirect measurement may allow bypassing problems that are inherent to quantification of subjective experience such as suggestion, variability due to beliefs and top-down influences (Rohde et al., 2011). Proprioceptive drift has been reported to correlate with the subjective feeling of ownership over the rubber hand (e.g., Botvinick and Cohen, 1998; Longo et al., 2008; Lopez et al., 2010). This drift has been widely used as a proxy for the subjective feeling of ownership. However, recent studies indicate that a dissociation between subjective ratings and drift exists (e.g., Shimada et al., 2009; Rohde et al., 2011; Romano et al., 2015). This dissociation implies that conclusions about the experience of ownership cannot be drawn from measuring proprioceptive drift alone. There is more or less a consensus in the field that objective measures such as proprioceptive drift or skin conductance response should be combined with subjective ratings (Rohde et al., 2011; Kalckert and Ehrsson, 2012; Asai, 2015).

Originally, Botvinick and Cohen (1998) proposed a bottom-up explanation of the RHI which suggests that synchronized tactile and visual stimulation are needed for the illusion to occur, since the illusion was not elicited with the use of asynchronous stimulation (Botvinick and Cohen, 1998; Ehrsson et al., 2004). However, intermodal matching of tactile and visual information does not seem sufficient to generate an experience of body ownership. Most studies use a rubber hand that visually corresponds to a human hand in shape, color and texture. If synchronous multisensory stimulation is the single factor causing body ownership, then it should be possible to induce ownership over objects that do not visually resemble a limb. Studies show that the illusion is not elicited when a neutral object such as a wooden plank is used instead of a rubber hand (Haggard, 2005; Holmes et al., 2006; Haans et al., 2008; Tsakiris et al., 2008, 2010b). Armel and Ramachandran (2003) report that a sense of ownership can be elicited over a table to some extent, but significantly less than over a rubber hand as measured by intensity ratings and skin conductance response (Armel and Ramachandran, 2003). Instead, a (partial) visual match between the object and the subject’s hand is usually needed for body ownership over the artificial hand. Also, the sense of body ownership is diminished when the rubber hand is of a different laterality or is placed in an incongruent anatomical posture in relation to the subject’s own hand (Pavani et al., 2000; Tsakiris et al., 2005; Costantini and Haggard, 2007). Apart from visual and postural congruence, spatial proximity (<30 cm) is another necessary condition for the illusion to occur (Lloyd, 2007).

When active control over objects is involved, however, people may experience some sense of ownership over non-corporeal objects. Ma and Hommel (2015) designed a virtual reality set-up, in which people could actively manipulate movement, size and color of either a virtual balloon or square. Participants reported a sense of ownership over the virtual effectors to some extent. The finding that people can experience ownership over non-corporeal objects would provide support for bottom-up approaches of self-representation (Botvinick and Cohen, 1998). Integration of temporally and spatially congruent multisensory signals would then be sufficient to induce the illusion of body ownership. However, the results of Ma and Hommel (2015) should be interpreted with caution, since participants reported low ownership ratings. Moreover, the ownership illusion was more convincing when the virtual effectors seemed to be connected to the participant’s body, suggesting that Gestalt principles influence self-perception. These Gestalt principles indicate that connectedness, governed by the laws of proximity and continuity, is central to perceiving unity (Ellis, 1999). Studies by Newport and Preston (2010) and Tieri et al. (2015) indeed show that virtually detaching the hand or finger in virtual reality, thereby disrupting continuity, abolishes body ownership of the proxy hand completely (Newport and Preston, 2010; Tieri et al., 2015).

These studies suggest that integration of visual, tactile, and proprioceptive information mediates ownership of single limbs. Petkova and Ehrsson (2008) investigated how ownership of individual body parts translates into the experience of owning a whole body. They used a “‘body-swap”’ illusion, in which people experienced a virtual body to be their own through visuo-tactile stimulation. Their findings suggest that the experience of owning an entire body is produced by neuronal populations that integrate multisensory information across body segments (Petkova and Ehrsson, 2008). In a following study, Petkova et al. (2011) found that the first person visual perspective poses a fundamental constraint on the full-body illusion. This supports the proposed model that the sense of body ownership relies on mechanisms for multisensory integration operating in body-part-centered reference frames (Ehrsson et al., 2004; Makin et al., 2008; Petkova and Ehrsson, 2008).

Patients with schizophrenia have been shown to experience the RHI more strongly (Peled et al., 2003) and faster (Peled et al., 2000) compared to healthy controls, as indicated by self-report questionnaires. These results have generally been explained as a decreased sense of body ownership, or less distinct self-other perception in schizophrenia due to deficits in multisensory integration. However, these studies used introspective reports (questionnaires) for both diagnosing schizophrenia and strength of the illusion, and the results could therefore merely reflect a response bias. Also, the study conducted by Peled et al. (2000) lacked a control condition. A possible general tendency to endorse bodily sensations in people with schizophrenia can therefore not be excluded.

In another study, Thakkar et al. (2011) investigated the RHI in schizophrenic patients compared to healthy controls with the use of both subjective (questionnaire) and objective (proprioceptive drift, autonomic response) measurements. Experience of the RHI was stronger during synchronous stimulation in both healthy controls and patients, indicated by both self-report and proprioceptive drift. Moreover, people with schizophrenia reported a stronger RHI than controls. In regard to the objective measures, Thakkar et al. (2011) found a larger proprioceptive drift in patients, but only with synchronous stimulation. This may indicate that proprioceptive drift and introspective reports represent distinct aspects of the illusion or that one of the measures does not adequately capture strength of experiencing the RHI (see Holmes et al., 2006; Shimada et al., 2009; Rohde et al., 2011; Romano et al., 2015). Another finding of the study is that temperature dropped in the stimulated hand during right hand stimulation whereas it increased in the unstimulated hand. Altogether, the authors conclude embodiment of the RHI to be stronger in schizophrenic patients than in healthy controls, in agreement with previous investigations (Peled et al., 2000, 2003). Psychosis-like symptoms in otherwise healthy individuals were correlated to greater illusion susceptibility/strength of body ownership in the RHI with synchronous stimulation in a study conducted by Germine et al. (2013).

The RHI is thought to arise from multisensory (visual and tactile) information overruling stored body representations (Tsakiris, 2010). Following this, stronger experience of the illusion could either result from increased multisensory integration, a stronger reliance on visual cues overriding proprioceptive information, or weaker pre-existing body representations. Previous research demonstrates that being prone to psychosis is related to a decrease in multisensory integration, as opposed to an increase (e.g., Asai et al., 2011; Germine et al., 2013). This makes increased visual-tactile integration a less likely explanation for the abovementioned findings. Rather, this can be interpreted for support of the hypothesis of weaker stored body or somatosensory representations. Previous studies have indeed already indicated that cognitive and perceptual deficits in schizophrenia are due to inadequate coupling of sensory information to pre-existing representations and environmental context, the details of which are beyond the scope of this review (but see: Schneider et al., 2002; Fletcher and Frith, 2009). Higher susceptibility to the illusion may therefore be due to a stronger reliance on multisensory information over potentially weaker stored body representations.

In conclusion, these studies indicate that simultaneous intermodal stimulation is necessary, but not sufficient for a sense of ownership to occur in the RHI. Spatial proximity, visual resemblance and postural correspondence are also important in eliciting a sense of body-ownership. When active manipulation is involved however, these prerequisites are attenuated, suggesting that a sense of agency may be important for a sense of body ownership to occur. This provides some support for the hypothesis of Short and Ward (2009), who postulate that ownership can be experienced over any object, irrespective of visual appearance, if predictable action outcomes follow the intentions of the agent (Short and Ward, 2009). In the absence of direct control, however, other factors, such as visual resemblance, spatial proximity and postural correspondence may become more important. With respect to schizophrenia, studies with the RHI suggest an enhanced illusion. While multisensory integration seems to be impaired in schizophrenia, this is likely to result in a reduced rather than increased RHI. Therefore, factors other than bottom-up multisensory processes, such as a weakened stored body representation, may contribute to a disturbed sense of body ownership.

## Temporal and Intentional Binding

Multisensory integration is generally seen as a crucial component in coherent bodily self-experience and sense of body ownership. As discussed above, disturbances in sensory processing that involve multisensory integration have been shown to exist in people with schizophrenia. However, other factors seem to play a role. Recent studies have investigated the temporal factors important for multisensory integration, with a focus on the “temporal binding window”, which is defined as the timespan within which stimuli from different modalities are perceptually bound. These studies have demonstrated that the window of temporal binding is distorted in various neurodevelopmental disorders, such as autism, dyslexia and schizophrenia. For example, Graham et al. (2014) investigated body ownership in schizophrenia patients divided into three groups, namely patients with current, past, and no history of passivity symptoms. They used a projected-hand illusion, in which a live video image of the participant’s hand is projected onto a video screen, with the real hand and the image of the hand being separated by 15 cm. Two delay conditions were present in the illusion; *synchronous* (<10 ms video delay) and *asynchronous* (an additional imposed 500 ms delay) feedback. A remarkable finding is that patients in the subgroup with current passivity symptoms did not show the typical reduction in body illusion with asynchronous feedback (with a 500 ms delay in visual feedback) as opposed to the synchronous condition. So, the clinical subgroup with passivity symptoms continued to experience ownership over the projected hand during asynchronous stimulation, whereas the other subgroups did not retain the sense of ownership in this condition (Graham et al., 2014). This may suggest that the window of temporal binding, which provides connections between the self and external stimuli, is “wider” in patients with current passivity symptoms. Consequently, they experience stimuli that are further apart in time, as co-occurring. However, this effect has not been replicated and more research into this particular factor is warranted.

Other evidence in support of this increased window of temporal binding is found in studies that report an impairment in time perception in people with schizophrenia (Elvevåg et al., 2003; Franck et al., 2005). Patients with passivity symptoms have been shown to exhibit aberrant cognitive and motor timing. Particularly, these studies have shown that these people perceive events as happening closer in time than they actually occurred (Blakemore et al., 2000; Foucher et al., 2007). This links to a related psychological phenomenon called “intentional binding”. Research conducted by Haggard et al. (2002) shows that, when a voluntary action is followed by an expected sensory consequence, a psychological binding effect causes events to be perceived closer in time, which contributes towards a sense of self-agency and self-recognition. This intentional binding effect involves the subjective perception that cause and effect are drawn together in perceived time (Haggard et al., 2002). Subjective, intentional experience of actions contributes to the inference of self-recognition (Haggard et al., 2002; Haggard, 2005; Engbert and Wohlschläger, 2007). Outcome expectations, sensory feedback and causal beliefs all partly influence binding (reviewed by Moore and Obhi, 2012).

Another phenomenon that is linked to the sense of agency is sensory attenuation, in which the sensory consequences of self-produced action are attenuated compared to externally generated events (Blakemore et al., 2000; Shergill et al., 2005). Both temporal binding and sensory attenuation can be modulated by cognitive expectations about either the outcome or source of an action (Moore et al., 2009; Desantis et al., 2011; Gentsch and Schütz-Bosbach, 2011). When expectations about the outcome of an action are induced in an experiment through showing the action outcome before the actual action performance, stronger temporal binding (Moore et al., 2009) and stronger sensory attenuation (Gentsch and Schütz-Bosbach, 2011) are reported. In an extensive review, van der Weiden et al. (2015) show that individuals diagnosed with schizophrenia have impairments in both motor prediction and cognitive processes (such as biased expectation of actions), which may lead to over-attribution of agency (see van der Weiden et al., 2015). In addition, research conducted by Renes et al. (2013) shows that, when the outcome of an action is implicitly primed, healthy people experience an increased sense of agency over actions, whereas schizophrenic patients do not (Renes et al., 2013).

The finding that the temporal binding window in schizophrenia is altered has important implications. Precision of internal timing is a crucial element in a variety of processes, including self-recognition and sensory–motor awareness (Haggard et al., 2003). Accurate temporal and intentional binding is important in forming causal mental relationships. This binding effect is thought to be modulated by top-down processes associated with motor intentions, and subjective experience of anticipated actions can therefore have distal effects on sensory-motor perception. Expectation of actions may thus also be involved bodily experience and ownership, but the precise role of anticipatory mechanisms still needs to be clarified.

## Predictive Mechanisms and Anticipation

As discussed above, expectation of actions may also play an important role in bodily experience. Additional evidence for this comes from a study conducted by Ferri et al. (2014), who created an edited version of the RHI in which they aimed to measure illusion susceptibility in the absence of the multisensory integration that occurs with the experience of touch. In this experiment, participants were instructed to observe the hand of the experimenter approaching, without touching, either a rubber hand or a piece of wood placed on a table in front of them. In healthy participants, expectation of touch due to the sight of a hand approaching the rubber hand is sufficient to elicit ownership over the rubber hand. Schizophrenia patients, however, reported a much lower sense of ownership over the rubber hand compared to healthy controls. Apparently, the mere expectation of touch is not sufficient for patients to experience the illusion. A possible explanation is that people with schizophrenia anticipate touch in a different way than healthy subjects do, or have a deficit in their predictive mechanisms of action (Ferri et al. (2014).

Voss et al. (2010) recorded subjective time estimates of a self-initiated voluntary action (a key press) followed by a sensory effect (a tone). When the voluntary action had a high probability of causing a tone, healthy volunteers showed a predictive shift of the perceptual estimate of the action towards the tone, whereas schizophrenia patients did not show this effect. This indicates that patients may lack a predictive component of action awareness. The deficit in predicting the relationship between action and effect was correlated with severity of positive psychotic symptoms, specifically delusions and hallucinations. Furthermore, individuals with schizophrenia showed an exaggerated retrospective binding between action and tone (Voss et al., 2010). Other studies also show impaired anticipation of the position of a moving stimulus in schizophrenia with smooth pursuit eye movements (e.g., Ross et al., 2000). Additionally, research on the *Simon effect*, the finding that manual responses are faster and more accurate when the stimulus appears on the same side as the responding hand (Simon and Wolf, 1963), also suggests that predictive mechanisms are dysfunctional in patients (Lalanne et al., 2012).

## Efferent Motor Signals

In the static RHI only tactile, visual and proprioceptive information are involved in evoking the feeling of ownership. During passive movement, information from skin receptors, muscle spindles, joint receptors and visual feedback provide additional kinesthetic information (Edin and Johansson, 1995; Proske and Gandevia, 2012). In active movement, this information is accompanied by efferent information from voluntary motor commands and the sensory predictions they produce (Bays and Wolpert, 2007). This is described in the “forward model” (Frith et al., 2000), which proposes that the sensory consequences of self-produced actions are predicted using internal information, such as efference copies of a motor command (Bell, 2001). By comparing the internal prediction with the sensory afference, one can differentiate between externally caused events and self-produced actions. In case of a match between the different sources of information, the salience of sensory information is diminished, and the afference is interpreted as the result of a self-produced action. When there is mismatch, the action is interpreted as being externally caused (Synofzik et al., 2008). Attenuation of self-produced stimulation occurs in healthy controls and psychotic patients without passivity symptoms or auditory hallucinations. By contrast, self-generated stimulation is not attenuated relative to externally produced stimulation in patients with passivity symptoms and/or auditory hallucinations (Blakemore et al., 2002; Shergill et al., 2005). This would support the proposal that such symptoms are associated with a deficit in the forward mechanism that normally predicts and cancels out self-produced actions.

During active movements, when efference information is involved, recognition of a body part or action is enhanced in healthy individuals (Daprati et al., 1997; Farrer et al., 2003; MacDonald and Paus, 2003; Tsakiris et al., 2005; Nahab et al., 2011). The importance of efferent signals and proprioception in self-recognition was investigated by Haggard (2005) with a self-recognition experiment in which healthy participants saw either their own hand or the experiment’s through video feedback, while their hand was passively moved either by the participants own left hand (active movement) or by the experimenter (passive movement). Participants were asked to judge whether the hand was their own. During passive movement, with only proprioceptive information available, self-recognition was at chance level. With the added efferent information available, when the participant actively made his own hand move, performance in the self-recognition task significantly increased, indicating that efferent information enhanced self-recognition in this task. Shimada et al. (2009) showed that threshold for the detection of mismatches (such as delayed visual feedback) is indeed lower during passive movement, suggesting improved discrimination ability when efferent information is available. According to the authors, these results mean that efferent signals give more important cues for self-recognition than just proprioception.

Synofzik et al. (2010) examined whether lower performance of schizophrenic patients in action attribution tasks is due to inaccurate predictions about the sensory consequences of self-produced action. Participants were asked to perform pointing movements in a virtual-reality setup in which the visual feedback of movements was rotated. Patients noticed the visual rotation at a higher rotation angle compared to controls, and the size of this angle was correlated to delusions of influence reported in these patients. In a second experiment, participants had to estimate their pointing direction in the presence of rotated video feedback. Estimates done by patients were more influenced by the feedback rotation and showed higher variability compared to controls. Moreover, during trials without visual feedback, in which estimates are completely dependent on internal action-related signals, the variability in estimates was likewise increased. These findings support the suggestion that the “comparator mechanism”, which relates internal and external cues (Frith, 2012), is impaired in schizophrenia due to elevated variation in internal predictions about the sensory consequences of action. Importantly, aside from greater variability in internal predictions, external information about self-produced actions (in this case visual feedback) influenced self-agency judgments to a greater extent in patients. The weighting of internal and external cues with respect to self-action may depend on the reliability of internal predictions (Synofzik et al., 2010). Imprecise or unreliable internal predictions may cause patients to depend more strongly on external information on self-action such as vision. This is in line with previous research showing that schizophrenia patients with delusions of control fail to make rapid error corrections based on awareness of discrepancies between intended and predicted limb positions (Malenka et al., 1982; Frith and Done, 1989), even though they have no difficulty correcting errors based on visual feedback about limb position (Frith and Done, 1989). This suggests that individuals with schizophrenia are deficient in their ability to monitor ongoing motor behavior on the basis of internal, self-generated cues (Daprati et al., 1997; Blakemore et al., 2000, 2002).

Although studies on self-recognition have contributed to our understanding of self-experience, these paradigms can provide only indirect evidence for the role of sensory and efferent cues in body ownership. The participant’s body-part is objectified as it is presented as an external object, for example projected on a screen (Tsakiris, 2010). The participant is asked to judge whether the action or body part belongs to the self. These experiments therefore involve explicit judgments rather than the feeling of body ownership and agency (see also Synofzik et al., 2008). Experimentation with the feeling of body-ownership becomes possible when multisensory stimulation is used to alter the experience of the body. The experience of ownership of a body (part) can then be present in one condition, and absent in another, as in the RHI (Tsakiris, 2010).

Tsakiris et al. (2006) studied the importance of efferent signals in an adjusted version of the RHI in which the participants performed both passive and active finger movements. With passive movement, the feeling of ownership was specifically localized to the moved finger. When actively moving a finger, however, ownership expanded over the whole hand. This suggests that efferent motor signals can integrate limbs into a wider awareness of the body. The authors argue that efferent motor signals enhance self-recognition by facilitating a “global subjective awareness” of body parts. A purely proprioceptive sense of body-ownership is local and fragmented, but the motor sense of agency seems to integrate different body parts into a coherent, global awareness of the body (Tsakiris et al., 2006).

In line with this, other studies have recently tried to examine whether added signals from passive and active movement are as important as visuo-tactile cues for developing a sense of body ownership. Most of these studies compared the strength of experiencing the RHI as elicited by active movements, passive movements, or visuo-tactile stimulation. Conflicting results have emerged from this research. Some studies indicate that movement enhances body ownership (Tsakiris et al., 2006; Dummer et al., 2009), some report no differences between movement and no-movement conditions (Kalckert and Ehrsson, 2014) and another study suggest that movement decreases ownership (Walsh et al., 2011). A recent study by Riemer et al. (2013) found no difference in the subjective strength of the ownership illusion when induced by active movements or visuotactile stimulation. However, the study by Riemer et al. (2013) found that the proprioceptive drift was stronger for the actively moving RHI compared to the classical version. Kalckert and Ehrsson (2012) reported higher subjective ownership ratings during active movements compared to passive movements (Kalckert and Ehrsson, 2012).

An interesting finding of the study conducted by Kalckert and Ehrsson (2014) was that, despite differences in available sensory and motor information between the three induction types, a very similar illusion was elicited. No differences were found along the passive movement, active movement and visuo-tactile stimulation conditions. This suggests that the RHI does not depend on specific types of sensory signals. Rather, the spatiotemporal relationship of the available signals seems to be important (Ehrsson, 2012). More evidence emerges from a study conducted Walsh et al. (2011), who used a moving RHI and anaesthetized the finger with lidocaine, thereby eliminating somatosensory information from the superficial skin. Nevertheless, in this situation with only proprioceptive and visual information available, participants experienced the illusion of ownership (Walsh et al., 2011). It seems that a match between any two independent sources of sensory information such as visual and tactile information or correlated sensorimotor signals can elicit the illusion. This explanation emphasizes bottom-up mechanisms in the illusion, but does not exclude top-down influences, which may pose* a priori* constraints on the types of objects can become part of one’s own body or may modulate the experience of ownership (Tsakiris et al., 2010a; Petkova et al., 2011). Moreover, Kalckert and Ehrsson (2014) found that active movements did not increase strength of the illusion, which does not support the proposal that efferent signals from voluntary motor commands are important for experiencing ownership (Kalckert and Ehrsson, 2014).

In order to understand how an enduring absence of movement-related signals affects body ownership, Burin et al. (2015) administered the classical RHI to a group of healthy participants and to a group of neurological patients affected by left upper limb hemiplegia without proprioceptive or tactile deficits. Their results show that patients experienced a stronger illusory effect when their left (affected) hand was stimulated, but the illusion was absent when the right (unaffected) hand was stimulated. This indicates that individuals with hemiplegia have a weaker/more flexible sense of body ownership for the affected hand, but an enhanced/more rigid one for the healthy hand (Burin et al., 2015). A prolonged absence of efferent signals may thus induce a more flexible sense of body ownership.

Overall, at present there is no consensus on the extent to which efferent signals contribute to the sense of body ownership. The discrepancy in the results obtained in above mentioned studies may be partly due to differences in the setups (model hand and projected hand), types movements that are used (full hand movements and finger movements) and the measures for illusion strength. Some studies only used proprioceptive drift (Tsakiris et al., 2006; Kammers et al., 2009) others only questionnaires (Dummer et al., 2009; Longo and Haggard, 2009) or a combination of measures but obtained discrepant results (Kalckert and Ehrsson, 2012; Riemer et al., 2013). It is therefore not clear to what extent efferent signals could contribute to the change in body ownership observed in schizophrenia. Self-recognition paradigms have shown us that individuals with schizophrenia exhibit elevated variation in internal predictions about the sensory consequences of action. Individuals with schizophrenia are deficient in their ability to monitor ongoing motor behavior on the basis of internal, self-generated cues (Blakemore et al., 2000, 2002). Moreover, external information about self-produced actions (in this case visual feedback) influences self-agency judgments to a greater extent in schizophrenia patients (Synofzik et al., 2010). These studies presented the participant’s body-part as an external object, for example projected on a screen (Tsakiris, 2010). It would be interesting to investigate the contribution of efferent signals to ownership experience specifically with the use of the moving RHI in schizophrenia.

## The Relationship Between Self-Agency and Body Ownership

For a long time, it was thought that patients with schizophrenia have a selective deficit in their sense of agency, whilst their sense of body ownership would be intact. This links to the idea that sense of agency and body-ownership are completely distinct phenomena, without any shared component (Longo et al., 2008). The model that stems from this view is termed the “independence model” (Tsakiris et al., 2010a). However, several lines of evidence now indicate that there could be a relationship between the sense of agency and body ownership. This idea is reflected in the “additive model”, in which the experience of self- agency consists of the sense of body-ownership, plus the added possible sense of voluntary control over actions (Longo and Haggard, 2009; Tsakiris et al., 2010a). In this view, the sense of agency and body ownership are strongly related to each other, and sense of agency is rooted in body ownership. Other relationships between agency and ownership are also possible, in which agency and ownership are dissociable components of bodily experience but do interact. Agency is not a necessary condition for body ownership as a sense of ownership over a rubber hand can be elicited without movement, but perceived non-agency may prevent ownership (Newport et al., 2010) while perceived agency may enhance the sense of body ownership (Tsakiris et al., 2006).

In an experiment utilizing functional magnetic resonance imaging (fMRI), Tsakiris et al. (2010a) investigated this relationship between the sense of body ownership and agency. They influenced the sense of body ownership by showing either real-time or delayed visual feedback of movement, whereas agency was manipulated through voluntary and passive movement. As shown before, synchronous visual feedback can cause body parts and bodily events to be attributed to oneself. Thus, the condition producing body-ownership would follow from passive movement with synchronous visual feedback, and the condition with active movement and synchronous visual feedback would result in a sense of agency. The additive model predicts that the fMRI shows common activation in areas in both the conditions that produce agency and body-ownership. Secondly, there should also be additional activation of area(s) for the condition producing agency. The independence model, on the other hand, predicts that the brain contains different networks for sense of body ownership and agency. Therefore, there should be no common activation in conditions inducing agency and ownership and distinct activations should be shown in the agency condition that are not seen in the other conditions. Also, specific activation should be seen in the condition of ownership that is absent from the agency condition.

Interestingly, the results of the introspective data (questionnaire) showed support of the additive model. Subjects described significantly more feelings of agency in the AS (active synchronous) condition, which causes a sense of agency, compared to the three remaining conditions. Moreover, participants reported stronger sense of body-ownership in the AS condition compared to the passive synchronous (body ownership) condition. This indicates that agency enhances the sense of body ownership. The fMRI data, on the other hand, showed support for the independence model. There was no common activation in brain areas in the condition producing both agency and body ownership. Also, both body-ownership and agency showed different and exclusive activation of brain areas, which provides evidence that different neural networks underlie these experiences. Tsakiris et al. (2010a) postulate that the discrepancy between neural activation and questionnaire data indicates that conscious experience and brain activity cannot be mapped one-to-one. Alternatively, this result may reveal a limitation of introspective data.

In an earlier study, Marcel (2003) differentiated between attribution of an action to one’s self, and attribution of the *intention* of the action to one’s self. For example, patients with an anarchic hand report the distinct sense of their involuntary movements as being their own, but they do not experience intending these movements. They do not have a feeling of agency, whilst they continue to experience body ownership (Marcel, 2003). Also, recognizing actions as one’s own requires an explicit judgment, contrary to the experience of an action as being one’s own. Introspective data may not properly distinguish the difference between ownership of intentional action and ownership of more general bodily actions and sensations (Tsakiris et al., 2010a). The reverse dissociation, in which people retain a sense of agency, but not body ownership, is harder to envisage. As Tsakiris et al. (2010a) point out, however, this can be seen in anosognosia patients with hemiplegia who also have somatoparaphrenic delusions. When a patient looks at her arm she would report that the arm belongs to another person. Nevertheless, the patient denies paralysis and describes that she is able to move her arm voluntarily, which indicates awareness of self-agency (Fotopoulou et al., 2008). This may indicate a double dissociation between agency and ownership.

On the other hand, there is also evidence to suggest that the sense self-agency and body ownership are interacting and overlapping rather than modular and discrete (Legrand, 2006; Synofzik et al., 2008). Action processes that contribute to a sense of agency depend on processes involved in body ownership, such as multisensory integration and internal body representations (Waters and Badcock, 2010). Lower performance on attribution tasks in people with schizophrenia have often been brought up as evidence for a selective dysfunction in sense of agency (Franck et al., 2001). It has been argued, however, that performance on these tasks, in which subjects have to judge whether a movement belongs to oneself based on visual feedback, requires “*an implicit knowledge of one’s body as a sensory object that is moving (i.e., a sense of body ownership)*” (Waters and Badcock, 2010). This means that attribution errors may partly be caused by a distortion in sense of body ownership. Moreover, deficits in integrating visual and somatosensory signals from limbs have been shown to affect the ability to judge self-action (Bulot et al., 2007).

Kalckert and Ehrsson (2012) used a version of the RHI in which they systematically varied the relative timing of the finger movements (synchronous vs. asynchronous), the mode of movement (active vs. passive), and the position of the model hand (anatomically congruent vs. incongruent positions). Their results indicate that voluntary finger movements elicit a robust illusion of owning the rubber hand. Asynchrony eliminated both ownership and agency, passive movements selectively eliminated the sense of agency, and incongruent positioning diminished ownership but not agency. These findings provide evidence for a double dissociation of ownership and agency. However, the authors also note that the sense of agency was stronger when the hand was perceived to be part of the participant’s body, and in this condition a significant correlation between agency and ownership was found. A later study by Kalckert and Ehrsson (2014) reported that questionnaire ratings of ownership and agency were correlated across individuals, even in the passive versions of the illusion (passive movement and visuo-tactile). This result suggests that ownership modulates agency and produces a weak tendency for agency even in the absence of intentions and voluntary motor commands (Kalckert and Ehrsson, 2014).

Garbarini et al. (2016) showed that people with schizophrenia exhibit a greater interference of visual information about the movements of another person on their sense of agency. The authors administered two versions of a manual drawing task to 20 schizophrenic patients and 20 age-matched healthy controls. In the bimanual version, participants had to draw lines with one hand and circles with the other. In the modified version, participants were instructed to draw lines while observing the examiner drawing circles from a first person perspective. In the bimanual version, patients and controls showed a comparable interference effect. In the observation version, however, schizophrenics showed a significantly greater interference the examiners’ hand drawing circles on their own hand drawing lines. This effect was significantly correlated to the strength of the positive symptoms (hallucinations and delusions) and to the sense of agency that was reported during the task. These findings suggest that an altered sense of agency can induce changes in the motor system. However, there was no correlation between motor performance and feeling of ownership over the experimenter’s hand (Garbarini et al., 2016).

Louzolo et al. (2015) used the version of the RHI that is based on finger movements rather than tactile stimulation (Kalckert and Ehrsson, 2012, 2014) to investigate the relationship between delusion-proneness and sense of agency. Individuals with a high delusion-proneness score gave equally strong agency ratings in active and passive conditions, suggesting that they experienced both conditions as self-produced (Louzolo et al., 2015). This may imply that delusion-prone individuals experience agency in the absence of motor intentions possibly due to increased reliance on external sensory signals, in this case vision. This was also reflected in the ownership scores. These results are in line with the idea that motor prediction is weakened (Blakemore et al., 2000; Blakemore and Frith, 2003; Shergill et al., 2005; Teufel et al., 2010) whereas external cues become more salient (Synofzik et al., 2010; Voss et al., 2010) in delusion-prone individuals. Hypersalient processing of both agency and ownership cues might be related to failures of self-recognition in schizophrenia (Waters and Badcock, 2010).

To summarize, the relationship between agency and ownership remains elusive. A double dissociation between the sense of agency and body ownership may exist, but more research on this subject in schizophrenia is warranted. Rather, phenomenological studies indicate that patients have distortions in the sense of self beyond only action awareness, extending over a broad area of self-experience, including the sense of body ownership. Previous studies with healthy participants and neurological patients suggest that agency and body ownership are dissociable to some extent, but also interact with agency affecting ownership and* vice versa*. Studies indicate that agency and ownership are dissociable elements of self-experience. However, evidence from healthy individuals also suggests that the sense of agency and ownership interact. The disturbed sense of agency in schizophrenia may therefore contribute to a disturbed sense of body ownership. Further experimental studies are necessary to disentangle agency and body ownership related problems in schizophrenia.

## Conclusion

In this article, we have reviewed the various sources of information that may contribute to a coherent sense of body ownership. It is thought that bodily experience is the result of a complex integration of both bottom-up sensory processes and higher order, top-down bodily and perceptual representations. Bodily experience involves the integration of multisensory information. Ownership is enhanced by visual capture of proprioceptive information on limb position. In order to infer body ownership, people with schizophrenia rely to a greater extent on external stimuli, such as visual information, that override weaker stored body representations. In addition, the temporal and intentional binding window seem to be altered in people with schizophrenia. This effect of temporal binding is thought to be modulated by top-down processes associated with motor intentions. Moreover, predictive, re-afferent information on the spatial position of body parts is related to increased sense of agency and ownership (Giummarra et al., 2008). We have also discussed evidence for a disturbance in predictive mechanisms that normally allow for anticipation of upcoming events.

The evidence presented in this review indicates that people with schizophrenia have higher variability in internal predictions about the sensory consequences of action. Importantly, aside from internal predictions, additional external information about self-produced actions influences self-agency judgments to a greater extent. The weighting of internal and external cues with respect to self-action may depend on the reliability of internal predictions (Synofzik et al., 2010). Imprecise or unreliable internal predictions might cause patients to depend more strongly on external information on self-action such as vision. People with schizophrenia and individuals with elevated psychosis-prone characteristics (Teufel et al., 2010), show reduced susceptibility to perceptual illusions, a phenomenon driven by prior expectations (reviewed in Notredame et al., 2014). The abnormalities in low-level processing that are observed in schizophrenia may indicate imprecise prior beliefs (Denève and Jardri, 2016). A possible mechanism could involve the capacity of higher-order areas to predict the state of lower level representations (sensory, motor, or cognitive). This is described in the predictive coding framework (Friston and Kiebel, 2009). The Bayesian model of schizophrenia proposes that stronger aberrant signals may contribute to even more imprecise expectations which may influence belief formation, which eventually results in delusions (Fletcher and Frith, 2009; Denève and Jardri, 2016). Imprecise or weaker predictions may result in increased salience of external cues due to a lack of suppression of the input signals. Alternatively, aberrant or hypersalient input signals may prevent establishment of stable low-level predictions (Schmack et al., 2013). Further examinations into the interaction between higher order cognitive processes and sensory-motor processes could prove to be crucial for understanding abnormal self-experience in schizophrenia. In an extensive review, van der Weiden et al. (2015) show that individuals diagnosed with schizophrenia have impairments in both motor prediction and cognitive processes, such as biased expectation of actions (van der Weiden et al., 2015).

We have provided arguments against the prevailing idea that people with schizophrenia have a selective deficit in the sense of agency. Even though concepts of agency and ownership can be dissociated to some extent, there is also evidence to suggest that the sense of agency and body ownership are interconnected. Evidence for this in healthy participants comes from a study conducted by Tsakiris et al. (2006). During active finger movement, the RHI extended to the whole hand. A purely proprioceptive sense of body-ownership is local and fragmented, but the motor sense of agency integrates different body parts into a coherent, global awareness of the body (Tsakiris et al., 2006). Other studies in healthy participants indicate that people are more likely to experience an external object as part of their body when they have active control over the object. Agency and ownership seem to be dissociable yet interacting, but there is no consensus on this topic yet.

In summary, studies so far show that individuals with schizophrenia have a disturbed sense of body ownership. This review discussed several factors that may contribute. They include deficits in multisensory integration, a weaker stored representation of the body, differences in temporal binding as well as impairments in predicting sensory consequences of efferent motor signals. The latter is particularly relevant for the reduced sense of agency in schizophrenia patients. While agency has been considered to be separate from the sense of body ownership, recent studies with healthy participants suggest that they are linked and that an enhanced sense of agency increases feelings of body ownership. The reduced sense of agency in schizophrenic patients may therefore be one of the contributing factors with regards to the disturbed sense of body ownership. Vice versa, a reduced sense of body ownership may also contribute to a reduced sense of agency. Future studies should explicitly target the precise relation between the two in schizophrenia.

## Author Contributions

All authors contributed extensively to the final version of this manuscript. HCD and MK both contributed to the initial idea and design. MK provided the first draft of the manuscript. HCD provided critical feedback and rewrote sections of the initial article. Both authors contributed to revision and editing of the final version. HCD and MK approve of the manuscript to be published.

## Conflict of Interest Statement

The authors declare that the research was conducted in the absence of any commercial or financial relationships that could be construed as a potential conflict of interest.

## References

[B204] Armel K. C. Ramachandran V. S. (2003). Projecting sensations to external objects: evidence from skin conductance response. Proc. Biol. Sci., 270, 1499–1506. 10.1098/rspb.2003.2364 12965016 PMC1691405

[B2] Arzy S. Overney L. S. Landis T. Blanke O. (2006). Neural mechanisms of embodiment: asomatognosia due to premotor cortex damage. Arch. Neurol. 63, 1022–1025. 10.1001/archneur.63.7.1022 16831974

[B3] Asai T. (2015). Agency elicits body-ownership: proprioceptive drift toward a synchronously acting external proxy. Exp. Brain Res. 234, 1163–1174. 10.1007/s00221-015-4231-y 25716612

[B4] Asai T. Mao Z. Sugimori E. Tanno Y. (2011). Rubber hand illusion, empathy and schizotypal experiences in terms of self-other representations. Conscious. Cogn. 20, 1744–1750. 10.1016/j.concog.2011.02.005 21371911

[B5] Bays P. M. Wolpert D. M. (2007). Computational principles of sensorimotor control that minimize uncertainty and variability. J. Physiol. 578, 387–396. 10.1113/jphysiol.2006.120121 17008369 PMC2075158

[B6] Bell C. C. (2001). Memory-based expectations in electrosensory systems. Curr. Opin. Neurobiol. 11, 481–487. 10.1016/s0959-4388(00)00238-5 11502396

[B210] Blakemore S. J. Frith C. (2003). Self-awareness and action. Curr. Opin. Neurobiol., 13, 219–224. 10.1016/S0959-4388(03)00043-6 12744977

[B8] Blakemore S. J. Smith J. Steel R. Johnstone E. C. Frith C. D. (2000). The perception of self-produced sensory stimuli in patients with auditory hallucinations and passivity experiences: evidence for a breakdown in self-monitoring. Psychol. Med. 30, 1131–1139. 10.1017/s0033291799002676 12027049

[B9] Blakemore S. J. Wolpert D. M. Frith C. D. (2002). Abnormalities in the awareness of action. Trends Cogn. Sci. 6, 237–242. 10.1016/s1364-6613(02)01907-1 12039604

[B10] Botvinick M. Cohen J. (1998). Rubber hands ‘feel’ touch that eyes see. Nature 391, 756–756. 10.1038/35784 9486643

[B11] Bulot V. Thomas P. Delevoye-Turrell Y. (2007). Sense of agency: experiencing is not judging. Encephale 33, 603–608. 10.1016/S0013-7006(07)92060-6 18033150

[B208] Burin D. Livelli A. Garbarini F. Fossataro C. Folegatti A. Gindri P.. (2015). Are movements necessary for the sense of body ownership? Evidence from the rubber hand illusion in pure hemiplegic patients. PLoS One, 10: e0117155. 10.1371/journal.pone.0117155 25775041 PMC4361688

[B12] Costantini M. Haggard P. (2007). The rubber hand illusion: sensitivity and reference frame for body ownership. Conscious. Cogn. 16, 229–240. 10.1016/j.concog.2007.01.001 17317221

[B13] Daprati E. Franck N. Georgieff N. Proust J. Pacherie E. Dalery J.. (1997). Looking for the agent: an investigation into consciousness of action and self-consciousness in schizophrenic patients. Cognition 65, 71–86. 10.1016/s0010-0277(97)00039-5 9455171

[B14] de Haan S. Fuchs T. (2010). The ghost in the machine: disembodiment in schizophrenia-two case studies. Psychopathology 43, 327–333. 10.1159/000319402 20664309

[B15] Denève S. Jardri R. (2016). Circular inference: mistaken belief, misplaced trust. Curr. Opin. Behav. Sci. 11, 40–48. 10.1016/j.cobeha.2016.04.001

[B16] Desantis A. Roussel C. Waszak F. (2011). On the influence of causal beliefs on the feeling of agency. Conscious. Cogn. 20, 1211–1220. 10.1016/j.concog.2011.02.012 21396831

[B17] Dummer T. Picot-Annand A. Neal T. Moore C. (2009). Movement and the rubber hand illusion. Perception 38, 271–280. 10.1068/p5921 19400435

[B18] Edin B. B. Johansson N. (1995). Skin strain patterns provide kinaesthetic information to the human central nervous system. J. Physiol. 487, 243–251. 10.1113/jphysiol.1995.sp020875 7473253 PMC1156613

[B207] Ehrsson H. H. (2012). “The concept of body ownership and its relation to multisensory integration,” in The New Handbook of Multisensory Processing, ed. Stein B. E. (Cambridge, MA: MIT Press).

[B206] Ehrsson H. H. Spence C. Passingham R. E. (2004). That’s my hand! Activity in premotor cortex reflects feeling of ownership of a limb. Science 305, 875–877. 10.1126/science.1097011 15232072

[B20] Ellis W. D. (1999). A Source Book of Gestalt Psychology. (Vol. 2), Abingdon, Oxon: Psychology Press.

[B21] Elvevåg B. McCormack T. Gilbert A. Brown G. D. Weinberger D. R. Goldberg T. E. (2003). Duration judgements in patients with schizophrenia. Psychol. Med. 33, 1249–1261. 10.1017/s0033291703008122 14580079

[B22] Engbert K. Wohlschläger A. (2007). Intentions and expectations in temporal binding. Conscious. Cogn. 16, 255–264. 10.1016/j.concog.2006.09.010 17113309

[B23] Farrer C. Franck N. Paillard J. Jeannerod M. (2003). The role of proprioception in action recognition. Conscious. Cogn. 12, 609–619. 10.1016/s1053-8100(03)00047-3 14656504

[B25] Ferri F. Costantini M. Salone A. Di Iorio G. Martinotti G. Chiarelli A. (2014). Upcoming tactile events and body ownership in schizophrenia. Schizophr. Res. 152, 51–57. 10.1016/j.schres.2013.06.026 23835002

[B26] Fletcher P. C. Frith C. D. (2009). Perceiving is believing: a Bayesian approach to explaining the positive symptoms of schizophrenia. Nat. Rev. Neurosci. 10, 48–58. 10.1038/nrn2536 19050712

[B27] Fotopoulou A. Tsakiris M. Haggard P. Vagopoulou A. Rudd A. Kopelman M. (2008). The role of motor intention in motor awareness: an experimental study on anosognosia for hemiplegia. Brain 131, 3432–3442. 10.1093/brain/awn225 18812442

[B28] Foucher J. R. Lacambre M. Pham B. T. Giersch A. Elliott M. A. (2007). Low time resolution in schizophrenia: lengthened windows of simultaneity for visual, auditory and bimodal stimuli. Schizophr. Res. 97, 118–127. 10.1016/j.schres.2007.08.013 17884350

[B29] Franck N. Farrer C. Georgieff N. Marie-Cardine M. Daléry J. d’Amato T.. (2001). Defective recognition of one’s own actions in patients with schizophrenia. Am. J. Psychiatry 158, 454–459. 10.1176/appi.ajp.158.3.454 11229988

[B30] Franck N. Posada A. Pichon S. Haggard P. (2005). Altered subjective time of events in schizophrenia. J. Nerv. Ment. Dis. 193, 350–353. 10.1097/01.nmd.0000161699.76032.09 15870620

[B31] Friston K. Kiebel S. (2009). Predictive coding under the free-energy principle. Philos. Trans. R. Soc. Lond. B Biol. Sci. 364, 1211–1221. 10.1098/rstb.2008.0300 19528002 PMC2666703

[B32] Frith C. (2012). Explaining delusions of control: the comparator model 20 years on. Conscious. Cogn. 21, 52–54. 10.1016/j.concog.2011.06.010 21802318

[B34] Frith C. D. Blakemore S. J. Wolpert D. M. (2000). Abnormalities in the awareness and control of action. Philos. Trans. R. Soc. Lond. B Biol. Sci. 355, 1771–1788. 10.1098/rstb.2000.0734 11205340 PMC1692910

[B33] Frith C. D. Done D. J. (1989). Experiences of alien control in schizophrenia reflect a disorder in the central monitoring of action. Psychol. Med. 19, 359–363. 10.1017/s003329170001240x 2762440

[B35] Garbarini F. Mastropasqua A. Sigaudo M. Rabuffetti M. Piedimonte A. Pia L.. (2016). Abnormal sense of agency in patients with schizophrenia: evidence from bimanual coupling paradigm. Front. Behav. Neurosci. 10:43. 10.3389/fnbeh.2016.00043 27014005 PMC4783405

[B36] Gentsch A. Schütz-Bosbach S. (2011). I did it: unconscious expectation of sensory consequences modulates the experience of self-agency and its functional signature. J. Cogn. Neurosci. 23, 3817–3828. 10.1162/jocn_a_00012 21452945

[B37] Germine L. Benson T. L. Cohen F. Hooker C. I. L. (2013). Psychosis-proneness and the rubber hand illusion of body ownership. Psychiatry Res. 207, 45–52. 10.1016/j.psychres.2012.11.022 23273611

[B38] Giummarra M. J. Gibson S. J. Georgiou-Karistianis N. Bradshaw J. L. (2008). Mechanisms underlying embodiment, disembodiment and loss of embodiment. Neurosci. Biobehav. Rev. 32, 143–160. 10.1016/j.neubiorev.2007.07.001 17707508

[B39] Graham K. T. Martin-Iverson M. T. Holmes N. P. Jablensky A. Waters F. (2014). Deficits in agency in schizophrenia and additional deficits in body image, body schema and internal timing, in passivity symptoms. Front. Psychiatry 5:126. 10.3389/fpsyt.2014.00126 25309460 PMC4159976

[B40] Haans A. Ijsselsteijn W. A. de Kort Y. A. (2008). The effect of similarities in skin texture and hand shape on perceived ownership of a fake limb. Body Image 5, 389–394. 10.1016/j.bodyim.2008.04.003 18650135

[B41] Haggard P. (2005). Conscious intention and motor cognition. Trends Cogn. Sci. 9, 290–295. 10.1016/j.tics.2005.04.012 15925808

[B42] Haggard P. Clark S. Kalogeras J. (2002). Voluntary action and conscious awareness. Nat. Neurosci. 5, 382–385. 10.1038/nn827 11896397

[B43] Haggard P. Martin F. Taylor-Clarke M. Jeannerod M. Franck N. (2003). Awareness of action in schizophrenia. Neuroreport 14, 1081–1085. 10.1097/01.wnr.0000073684.00308.c0 12802207

[B44] Holmes N. P. Snijders H. J. Spence C. (2006). Reaching with alien limbs: visual exposure to prosthetic hands in a mirror biases proprioception without accompanying illusions of ownership. Percept. Psychophys. 68, 685–701. 10.3758/bf03208768 16933431 PMC1564193

[B45] Kalckert A. Ehrsson H. H. (2012). Moving a rubber hand that feels like your own: a dissociation of ownership and agency. Front. Hum. Neurosci. 6:40. 10.3389/fnhum.2012.00040 22435056 PMC3303087

[B46] Kalckert A. Ehrsson H. H. (2014). The moving rubber hand illusion revisited: comparing movements and visuotactile stimulation to induce illusory ownership. Conscious. Cogn. 26, 117–132. 10.1016/j.concog.2014.02.003 24705182

[B209] Kammers M. P. M. de Vignemont F. Verhagen L. Dijkerman H. C. (2009). The rubber hand illusion in action. Neuropsychologia 47, 204–211. 10.1016/j.neuropsychologia.2008.07.028 18762203

[B201] Kant I. Smith N. K. (2003). Critique of Pure Reason, Revised and Translated by Max Müeller. New York: Macmillan (originally published in 1781). 14769232

[B47] Lalanne L. van Assche M. Giersch A. (2012). When predictive mechanisms go wrong: disordered visual synchrony thresholds in schizophrenia. Schizophr. Bull. 38, 506–513. 10.1093/schbul/sbQ127 20876220 PMC3330002

[B48] Legrand D. (2006). The bodily self: the sensori-motor roots of pre-reflective self-consciousness. Phenomenol. Cogn. Sci. 5, 89–118. 10.1007/s11097-005-9015-6

[B49] Lloyd D. M. (2007). Spatial limits on referred touch to an alien limb may reflect boundaries of visuo-tactile peripersonal space surrounding the hand. Brain Cogn. 64, 104–109. 10.1016/j.bandc.2006.09.013 17118503

[B50] Longo M. R. Haggard P. (2009). Sense of agency primes manual motor responses. Perception 38, 69–78. 10.1068/p6045 19323137

[B205] Longo M. R. Schüür F. Kammers M. P. Tsakiris M. Haggard P. (2008). What is embodiment? A psychometric approach. Cognition 107, 978–998. 10.1016/j.cognition.2007.12.004 18262508

[B51] Lopez C. Lenggenhager B. Blanke O. (2010). How vestibular stimulation interacts with illusory hand ownership. Conscious. Cogn. 19, 33–47. 10.1016/j.concog.2009.12.003 20047844

[B202] Louzolo A. Kalckert A. Petrovic P. (2015). When passive feels active-delusion-proneness alters self-recognition in the moving rubber hand illusion. PLoS One, 10: e0128549. 10.1371/journal.pone.0128549 26090797 PMC4474665

[B52] Ma K. Hommel B. (2015). Body-ownership for actively operated non-corporeal objects. Conscious. Cogn. 36, 75–86. 10.1016/j.concog.2015.06.003 26094223

[B53] MacDonald P. A. Paus T. (2003). The role of parietal cortex in awareness of self-generated movements: a transcranial magnetic stimulation study. Cereb. Cortex 13, 962–967. 10.1093/cercor/13.9.962 12902395

[B54] Makin T. R. Holmes N. P. Ehrsson H. H. (2008). On the other hand: dummy hands and peripersonal space. Behav. Brain Res. 191, 1–10. 10.1016/j.bbr.2008.02.041 18423906

[B55] Malenka R. C. Angel R. W. Hampton B. Berger P. A. (1982). Impaired central error-correcting behavior in schizophrenia. Arch. Gen. Psychiatry 39, 101–107. 10.1001/archpsyc.1982.04290010073013 6119966

[B56] Marcel A. (2003). “The sense of agency: awareness and ownership of action,” in Agency and Self-Awareness, eds Roessler J. Eilan N. (Oxford, UK: Oxford University Press), 48–93.

[B57] Merleau-Ponty M. (1945). Phénomenologie de la Perception. Paris: Gallimard.

[B59] Moore J. W. Lagnado D. Deal D. C. Haggard P. (2009). Feelings of control: contingency determines experience of action. Cognition 110, 279–283. 10.1016/j.cognition.2008.11.006 19110240

[B58] Moore J. W. Obhi S. S. (2012). Intentional binding and the sense of agency: a review. Conscious. Cogn. 21, 546–561. 10.1016/j.concog.2011.12.002 22240158

[B60] Nahab F. B. Kundu P. Gallea C. Kakareka J. Pursley R. Pohida T.. (2011). The neural processes underlying self-agency. Cereb. Cortex 21, 48–55. 10.1093/cercor/bhq059 20378581 PMC3000563

[B61] Nelson B. Parnas J. Sass L. A. (2014). Disturbance of minimal self (ipseity) in schizophrenia: clarification and current status. Schizophr. Bull. 40, 479–482. 10.1093/schbul/sbu034 24619534 PMC3984529

[B63] Newport R. Pearce R. Preston C. (2010). Fake hands in action: embodiment and control of supernumerary limbs. Exp. Brain Res. 204, 385–395. 10.1007/s00221-009-2104-y 20012536 PMC2895889

[B62] Newport R. Preston C. (2010). Pulling the finger off disrupts agency, embodiment and peripersonal space. Perception 39, 1296–1298. 10.1068/p6742 21125957

[B64] Notredame C. E. Pins D. Deneve S. Jardri R. (2014). What visual illusions teach us about schizophrenia. Front. Integr. Neurosci. 8:63. 10.3389/fnint.2014.00063 25161614 PMC4130106

[B65] Pavani F. Spence C. Driver J. (2000). Visual capture of touch: out-of-the-body experiences with rubber gloves. Psychol. Sci. 11, 353–359. 10.1111/1467-9280.00270 11228904

[B66] Peled A. Pressman A. Geva A. B. Modai I. (2003). Somatosensory evoked potentials during a rubber-hand illusion in schizophrenia. Schizophr. Res. 64, 157–163. 10.1016/s0920-9964(03)00057-4 14613680

[B67] Peled A. Ritsner M. Hirschmann S. Geva A. B. Modai I. (2000). Touch feel illusion in schizophrenic patients. Biol. Psychiatry 48, 1105–1108. 10.1016/s0006-3223(00)00947-1 11094144

[B68] Petkova V. I. Ehrsson H. H. (2008). If I were you: perceptual illusion of body swapping. PLoS One 3:e3832. 10.1371/journal.pone.0003832 19050755 PMC2585011

[B69] Petkova V. I. Khoshnevis M. Ehrsson H. H. (2011). The perspective matters! Multisensory integration in ego-centric reference frames determines full-body ownership. Front. Psychol. 2:35. 10.3389/fpsyg.2011.00035 21687436 PMC3108400

[B70] Piaget J. (1954). The Construction of Reality in the Child (M. Cook, Trans.). New York, NY: Basic Books.

[B71] Proske U. Gandevia S. C. (2012). The proprioceptive senses: their roles in signaling body shape, body position and movement and muscle force. Physiol. Rev. 92, 1651–1697. 10.1152/physrev.00048.2011 23073629

[B203] Renes R. A. Vermeulen L. Kahn R. S. Aarts H. van Haren N. E. (2013). Abnormalities in the establishment of feeling of self-agency in schizophrenia. Schizophr. Res. 143, 50–54. 10.1016/j.schres.2012.10.024 23178108

[B72] Riemer M. Kleinböhl D. Hölzl R. Trojan J. (2013). Action and perception in the rubber hand illusion. Exp. Brain Res. 229, 383–393. 10.1007/s00221-012-3374-3 23307154

[B74] Rohde M. Di Luca M. Ernst M. O. (2011). The rubber hand illusion: feeling of ownership and proprioceptive drift do not go hand in hand. PLoS One 6:e21659. 10.1371/journal.pone.0021659 21738756 PMC3125296

[B75] Romano D. Caffa E. Hernandez-Arieta A. Brugger P. Maravita A. (2015). The robot hand illusion: inducing proprioceptive drift through visuo-motor congruency. Neuropsychologia 70, 414–420. 10.1016/j.neuropsychologia.2014.10.033 25446964

[B77] Ross R. G. Olincy A. Harris J. G. Sullivan B. Radant A. (2000). Smooth pursuit eye movements in schizophrenia and attentional dysfunction: adults with schizophrenia, ADHD and a normal comparison group. Biol. Psychiatry 48, 197–203. 10.1016/s0006-3223(00)00825-8 10924662

[B78] Sass L. A. Parnas J. (2003). Schizophrenia, consciousness and the self. Schizophr. Bull. 29, 427–444. 10.1093/oxfordjournals.schbul.a007017 14609238

[B211] Schmack K. Gòmez-Carrillo de Castro A. Rothkirch M. Sekutowicz M. Rössler H. Haynes J. D.. (2013). Delusions and the role of beliefs in perceptual inference. J. Neurosci. 33, 13701–13712. 10.1523/jneurosci.1778-13.2013 23966692 PMC6618656

[B79] Schneider U. Borsutzky M. Seifert J. Leweke F. M. Huber T. J. Rollnik J. D.. (2002). Reduced binocular depth inversion in schizophrenic patients. Schizophr. Res. 53, 101–108. 10.1016/s0920-9964(00)00172-9 11728843

[B80] Shergill S. S. Samson G. Bays P. M. Frith C. D. Wolpert D. M. (2005). Evidence for sensory prediction deficits in schizophrenia. Am. J. Psychiatry 162, 2384–2386. 10.1176/appi.ajp.162.12.2384 16330607

[B81] Shimada S. Fukuda K. Hiraki K. (2009). Rubber hand illusion under delayed visual feedback. PLoS One 4:e6185. 10.1371/journal.pone.0006185 19587780 PMC2702687

[B82] Short F. Ward R. (2009). Virtual limbs and body space: critical features for the distinction between body space and near-body space. J. Exp. Psychol. Hum. Percept. Perform. 35, 1092–1103. 10.1037/a0015873 19653751

[B83] Simon J. R. Wolf J. D. (1963). Choice reaction time as a function of angular stimulus-response correspondence and age. Ergonomics 6, 99–105. 10.1080/00140136308930679

[B84] Synofzik M. Thier P. Leube D. T. Schlotterbeck P. Lindner A. (2010). Misattributions of agency in schizophrenia are based on imprecise predictions about the sensory consequences of one’s actions. Brain 133, 262–271. 10.1093/brain/awp291 19995870

[B85] Synofzik M. Vosgerau G. Newen A. (2008). Beyond the comparator model: a multifactorial two-step account of agency. Conscious. Cogn. 17, 219–239. 10.1016/j.concog.2007.03.010 17482480

[B86] Teufel C. Kingdon A. Ingram J. N. Wolpert D. M. Fletcher P. C. (2010). Deficits in sensory prediction are related to delusional ideation in healthy individuals. Neuropsychologia 48, 4169–4172. 10.1016/j.neuropsychologia.2010.10.024 20974159 PMC3142618

[B87] Thakkar K. N. Nichols H. S. McIntosh L. G. Park S. (2011). Disturbances in body ownership in schizophrenia: evidence from the rubber hand illusion and case study of a spontaneous out-of-body experience. PLoS One 6:e27089. 10.1371/journal.pone.0027089 22073126 PMC3205058

[B88] Tieri G. Tidoni E. Pavone E. F. Aglioti S. M. (2015). Mere observation of body discontinuity affects perceived ownership and vicarious agency over a virtual hand. Exp. Brain Res. 233, 1247–1259. 10.1007/s00221-015-4202-3 25618006

[B89] Tsakiris M. (2010). My body in the brain: a neurocognitive model of body-ownership. Neuropsychologia 48, 703–712. 10.1016/j.neuropsychologia.2009.09.034 19819247

[B93] Tsakiris M. Longo M. R. Haggard P. (2010a). Having a body versus moving your body: neural signatures of agency and body-ownership. Neuropsychologia 48, 2740–2749. 10.1016/j.neuropsychologia.2010.05.021 20510255

[B90] Tsakiris M. Carpenter L. James D. Fotopoulou A. (2010b). Hands only illusion: multisensory integration elicits sense of ownership for body parts but not for non-corporeal objects. Exp. Brain Res. 204, 343–352. 10.1007/s00221-009-2039-3 19820918

[B91] Tsakiris M. Costantini M. Haggard P. (2008). The role of the right temporo-parietal junction in maintaining a coherent sense of one’s body. Neuropsychologia 46, 3014–3018. 10.1016/j.neuropsychologia.2008.06.004 18601939

[B92] Tsakiris M. Haggard P. Franck N. Mainy N. Sirigu A. (2005). A specific role for efferent information in self-recognition. Cognition 96, 215–231. 10.1016/j.cognition.2004.08.002 15996559

[B94] Tsakiris M. Prabhu G. Haggard P. (2006). Having a body versus moving your body: how agency structures body-ownership. Conscious. Cogn. 15, 423–432. 10.1016/j.concog.2005.09.004 16343947

[B95] Tsakiris M. Schütz-Bosbach S. Gallagher S. (2007). On agency and body-ownership: phenomenological and neurocognitive reflections. Conscious. Cogn. 16, 645–660. 10.1016/j.concog.2007.05.012 17616469

[B97] van der Weiden A. Prikken M. van Haren N. E. (2015). Self-other integration and distinction in schizophrenia: a theoretical analysis and a review of the evidence. Neurosci. Biobehav. Rev. 57, 220–237. 10.1016/j.neubiorev.2015.09.004 26365106

[B98] Voss M. Moore J. Hauser M. Gallinat J. Heinz A. Haggard P. (2010). Altered awareness of action in schizophrenia: a specific deficit in predicting action consequences. Brain 133, 3104–3112. 10.1093/brain/awQ142 20685805

[B99] Walsh L. D. Moseley G. L. Taylor J. L. Gandevia S. C. (2011). Proprioceptive signals contribute to the sense of body ownership. J. Physiol. 589, 3009–3021. 10.1113/jphysiol.2011.204941 21521765 PMC3139083

[B100] Waters F. A. Badcock J. C. (2010). First-rank symptoms in schizophrenia: reexamining mechanisms of self-recognition. Schizophr. Bull. 36, 510–517. 10.1093/schbul/sbn112 18753307 PMC2879682

